# The detailed transseptal puncture technique for optimal closure in patients with a patent foramen ovale

**DOI:** 10.3389/fcvm.2024.1453459

**Published:** 2024-11-29

**Authors:** Erdogan Ilkay, Ersin Sariçam, Fehmi Kaçmaz, Aysel Yakici, Çiğdem Koca, Özcan Özeke, Melike Polat, Murat Can Güney, Bilge Duran Karaduman, Mehmet Akif Erdöl, Mehmet Zulkuf Onal

**Affiliations:** ^1^ Cardiology Clinic, Liv Hospital, Ankara, Türkiye; ^2^ Medicana Ankara Hospital, Ankara, Türkiye; ^3^ Cardiology Clinic, Medicana Ankara Hospital, Ankara, Türkiye; ^4^ School of Medicine, Atilim University, Ankara, Türkiye; ^5^ Cardiology Clinic, School of Medicine, Üsküdar University, Istanbul, Türkiye; ^6^ Istanbul Haseki Educational Hospital, Istanbul, Türkiye; ^7^ School of Medicine, Yeditepe University, Istanbul, Türkiye; ^8^ Cardiology Clinic, Saglik Bilimleri University, Ankara, Türkiye; ^9^ Neurology Clinic, Medicana Ankara Hospital, Ankara, Türkiye

**Keywords:** patent foramen ovale, transseptal puncture, residual shunts, transseptal access, optimal closure schedule

## Abstract

**Background:**

The closure of a patent foramen ovale (PFO) using transseptal puncture has particular advantages and disadvantages. Thus, transseptal puncture should be re-evaluated in detail.

**Aims:**

We aimed to assess the effectiveness of the detailed transseptal puncture technique in patients who underwent PFO closure due to cryptogenic stroke or transient ischemic attack in terms of residual shunts and atrial fibrillation.

**Methods:**

We prospectively analyzed 144 consecutive patients who underwent PFO closure by the detailed transseptal puncture technique between February 2013 and April 2023 in two centers. All of the patients had a >10 mm long-tunnel PFO.

**Results:**

The procedural success rate was 100%. However, after the procedure, moderate pericardial effusion developed in one patient (0.7%) and an acute pulmonary embolism related to femoral vein thrombosis was observed in one patient (0.7%) during the first month. Complications related to the procedure were noted in two patients (1.4%) during the first month of follow-up. Residual shunts were observed in 1.4% of cases after PFO closure.

**Conclusion:**

We demonstrated that the detailed transseptal technique is safe and effective for PFO closure. The detailed transseptal PFO closure technique significantly reduced the risk of atrial fibrillation, and the occurrence of residual shunts was significantly low following the closure.

## Introduction

Transcatheter closure in patients who had suffered a cryptogenic stroke and have a patent foramen ovale (PFO) has been increasingly performed in the majority of experienced heart centers with high success rates, excellent long-term outcomes, and low complication rates (1, 2). Successful outcomes are closely linked to the complete closure of the PFO defect. Currently, two techniques are described for transcatheter closure of PFO. The first is the tunnel technique commonly used to pass the device through the PFO tunnel (3). The second is the transseptal technique, in which the septum primum is punctured to create a new hole and the device is passed through this artificial defect. The transseptal technique has been proposed for patients with a long-tunnel PFO or an uncrossable PFO (4–6).

Unfortunately, transcatheter closure procedures may result in certain complications, such as new-onset atrial fibrillation (AF) and a residual shunt (RS). The incidence of new-onset AF is 3%–14% in patients after PFO closure (7). Recurrent stroke and transient ischemic attack (TIA) have been observed in some patients due to an RS (8). In clinical practice, a residual shunt has been observed in up to 25% of patients after a PFO closure (9).

Incomplete closure of PFO should be taken into consideration in the management of recurrent stroke patients (9). Therefore, the transcatheter closure technique could be important in preventing an RS and recurrent stroke. There are only a small number of studies that have weighed the clinical outcomes of the transseptal technique against those of the tunnel technique in patients with PFO. Compared to the tunnel technique, the transseptal puncture technique in previous studies was shown to have a higher incidence of residual shunts and ischemic events (4, 5). However, one recent study showed that the long-term clinical outcomes of untreated PFO patients (uncrossable) who only received medical therapy were similar to those of the PFO closure patients who were treated with the tunnel technique (6).

The aim of this study was to re-evaluate the transseptal technique (detailed transseptal) in patients with a long-tunnel PFO in terms of more detailed anatomical measurements under transesophageal echocardiography (TEE) guidance and with a larger patient population. Moreover, the study prospectively investigated the long-term association of a residual PFO shunt with stroke or TIA and AF after PFO closure.

## Methods

This prospective cohort study included 144 patients (83 women, 61 men; mean age 40 ± 10.5 years) who underwent percutaneous transcatheter PFO closure with the transseptal technique due to cryptogenic stroke or TIA between February 2013 and April 2023 in two centers. All patients were informed and written informed consent was obtained from each patient. Conducted in accordance with the principles of the Declaration of Helsinki, the study was approved by the Human Research Ethics Committee at Medicana International Ankara Hospital (BSH 2018/06).

### Patient population

All the patients underwent comprehensive preprocedural evaluations, including transthoracic echocardiography (TTE) and TEE. All the patients were evaluated to determine if any other causes of stroke or ischemic attack were present. Atrial fibrillation was ruled out in all patients. All the patients were found to have a right-to-left shunt via contrast echocardiography. A PFO-induced right-to-left shunt was identified when agitated saline contrast appeared in the left atrium (LA) within three cardiac cycles in patients with right atrial opacification during the Valsalva maneuver or normal respiration (3). Shunting was defined as grade 1 when 3–9 contrast bubbles appeared, grade 2 when 10–30 contrast bubbles appeared, and grade 3 when more than 30 contrast bubbles appeared in the LA (8). Grades 2 and 3 were defined as high-grade shunts (4, 10). All of the patients had a long-tunnel PFO (≥10 mm) and high-grade shunts. Previous stroke history was present in 90 patients (62%) and a history of TIA was present in 54 patients (38%).

The exclusion criteria were the presence of left ventricular dysfunction, severe pulmonary artery hypertension, left atrial appendix, or other cardiac chamber thrombi. Severe pulmonary artery hypertension was diagnosed when peak pulmonary artery pressure exceeded 70% of systemic systolic blood pressure.

### Transesophageal echocardiography

First, all the patients underwent TTE to evaluate their interatrial septum and other cardiac functions. TEE was performed for all the patients before the procedure to detect the shape of PFO, tunnel length, the presence of a left-to-right shunt, and any accompanying atrial septal defect (ASD) or other congenital defects.

### PFO closure with the transseptal technique

Percutaneous transcatheter closure was performed under TTE or TEE guidance, and premedication with unfractionated heparin was administered with a dose of 100 IU/kg. In addition, optimal dual-antiplatelet therapy was given to all the patients during the preprocedural period. After the placement of the right femoral vein sheath, a 0.032 in guide wire was positioned into the superior vena cava. Under TEE guidance, the Mullins sheath was pulled back until it fell to the fossa ovalis. To cross the interatrial septum at the optimal point, the junction point of the septum primum and septum secundum was punctured using an 8 Fr standard Mullins transseptal sheath and a Brockenbrough needle. After crossing the interatrial septum, a dilatator was moved into the LA and a crimped wire was moved into the LA through the dilatator.

A long introducer sheath was then inserted over the wire and the device was placed using the standard technique. First, the left side disc was released and the whole system was pulled back till the left atrial disc contacted the septum, and then the right atrial disc was released.

The position and stability of the device were checked using the Minnesota maneuver. Finally, the device was fully released.

Two types of devices were used in our study: the Amplatzer PFO Occluder (Abbott, Chicago, IL, USA) and the Figulla Flex II PFO Occluder (Occlutech GmbH, Jena, Germany).

### The detailed transseptal technique and device selection

Previous studies utilized the classic transseptal PFO closure technique, relying on single-plane measurements. However, we re-evaluated this approach using bicaval and anterior-posterior (A-P) imaging to assess the tunnel entry and exit in two projections via TEE imaging. The puncture point was identified based on both projections, located at the junction of the septum primum and septum secundum. We measured the distance between this puncture point and the PFO's opening into the left atrium. To ensure complete coverage of the PFO, the device size was selected according to these measurements. This technique offers the potential to reduce the likelihood of a residual shunt following PFO closure.

**Figure 1:**
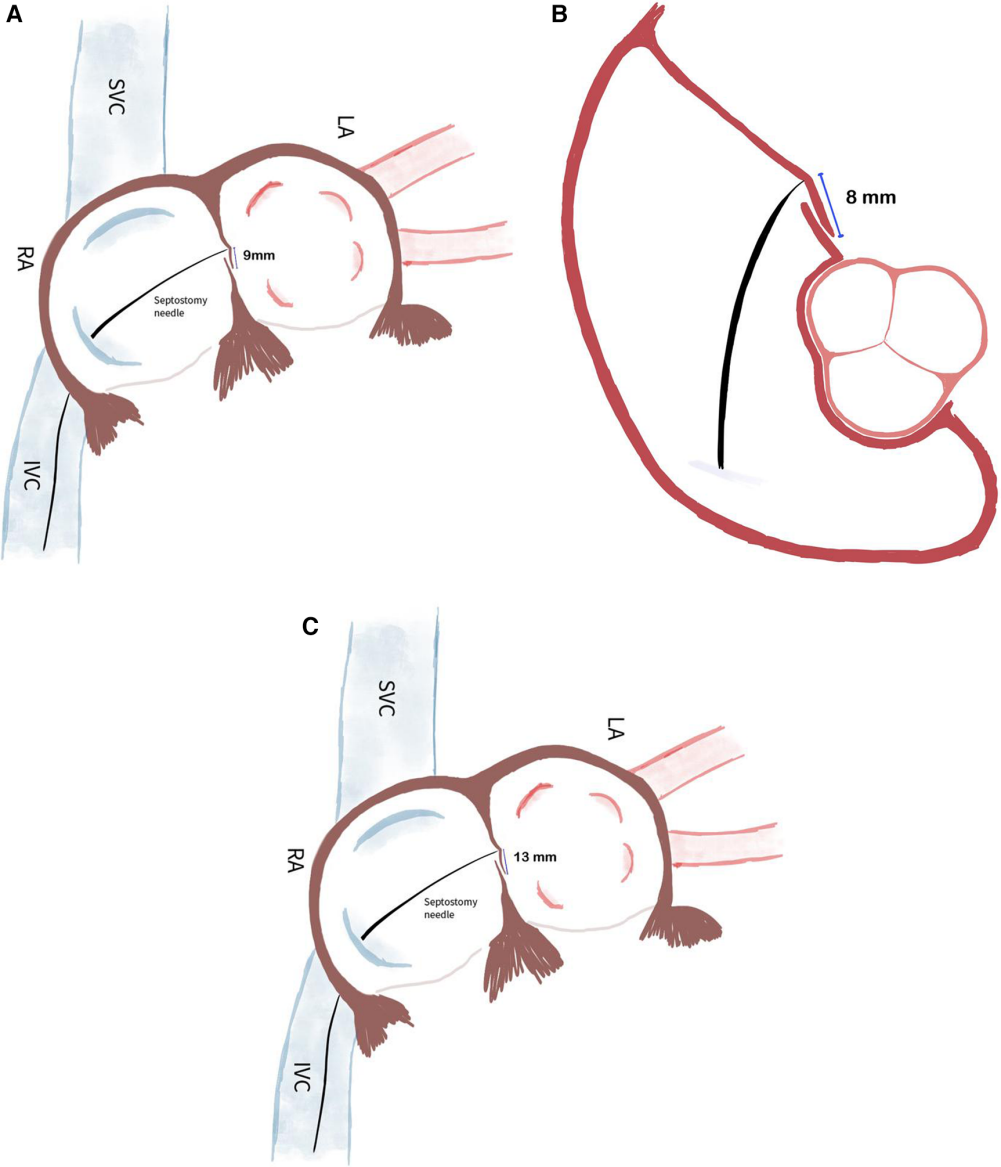
**(A)** When the septostomy point was placed lower (9 mm), the correct device selection was made possible without increasing the device size. If the device had been selected by taking images from a single section (A-P), a too-small device would have been selected and a residual shunt would have been left. **(B)** In the anterior-posterior section, the distance between the septostomy point and the tip of the PFO is 8 mm. **(C)** In the bicaval section of the same patient, this distance is 13 mm.

**Figure 2:**
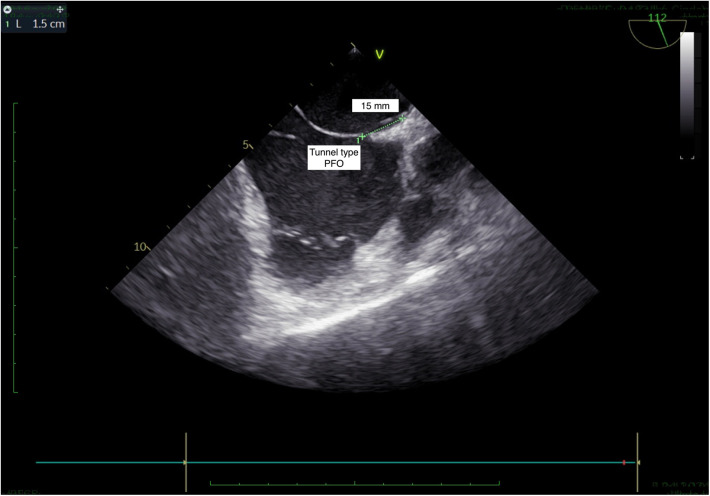
TEE, bicaval view: tunnel type PFO.

**Figure 3:**
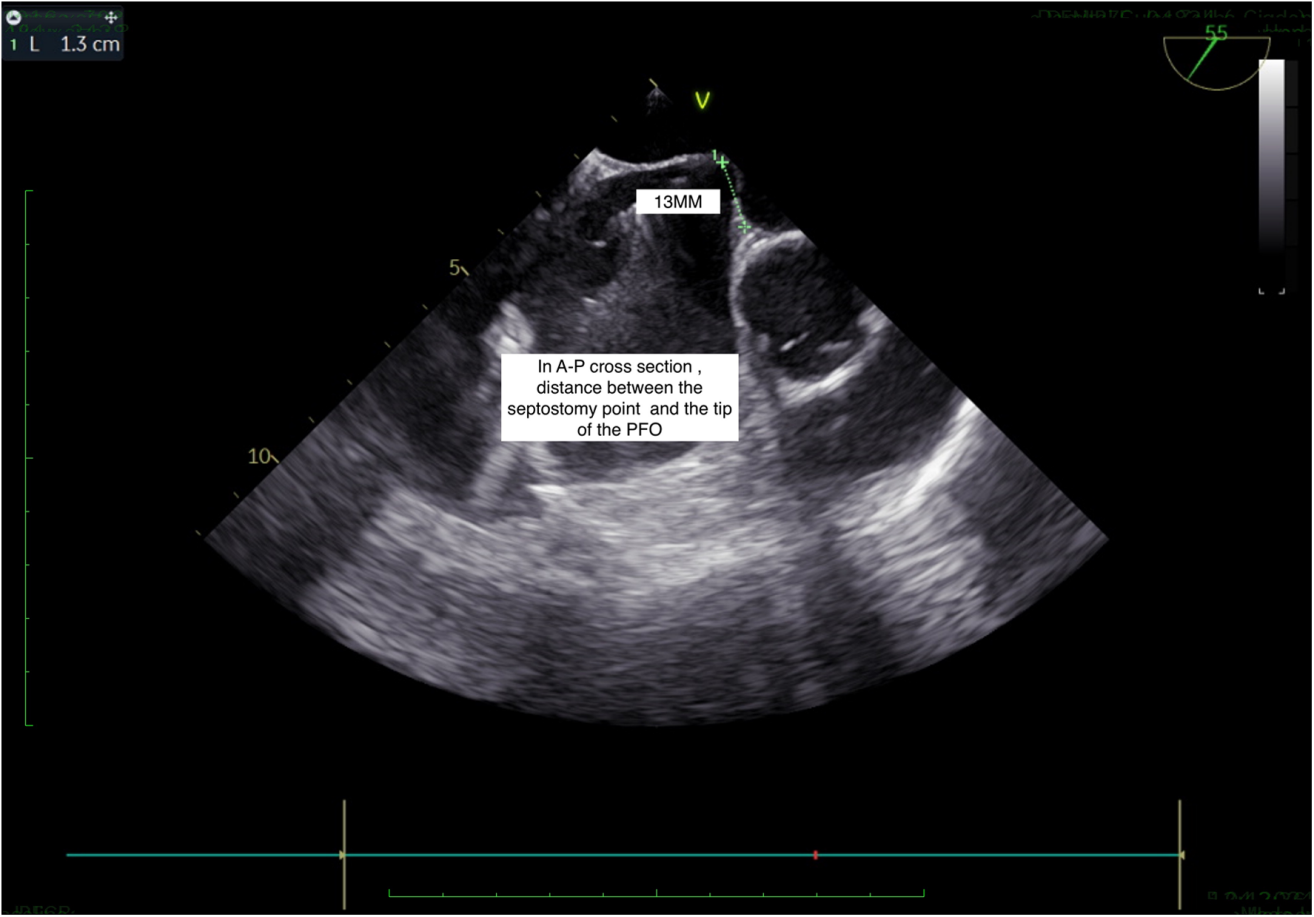
TEE, short axis view: septostomy distance.

**Figure 4:**
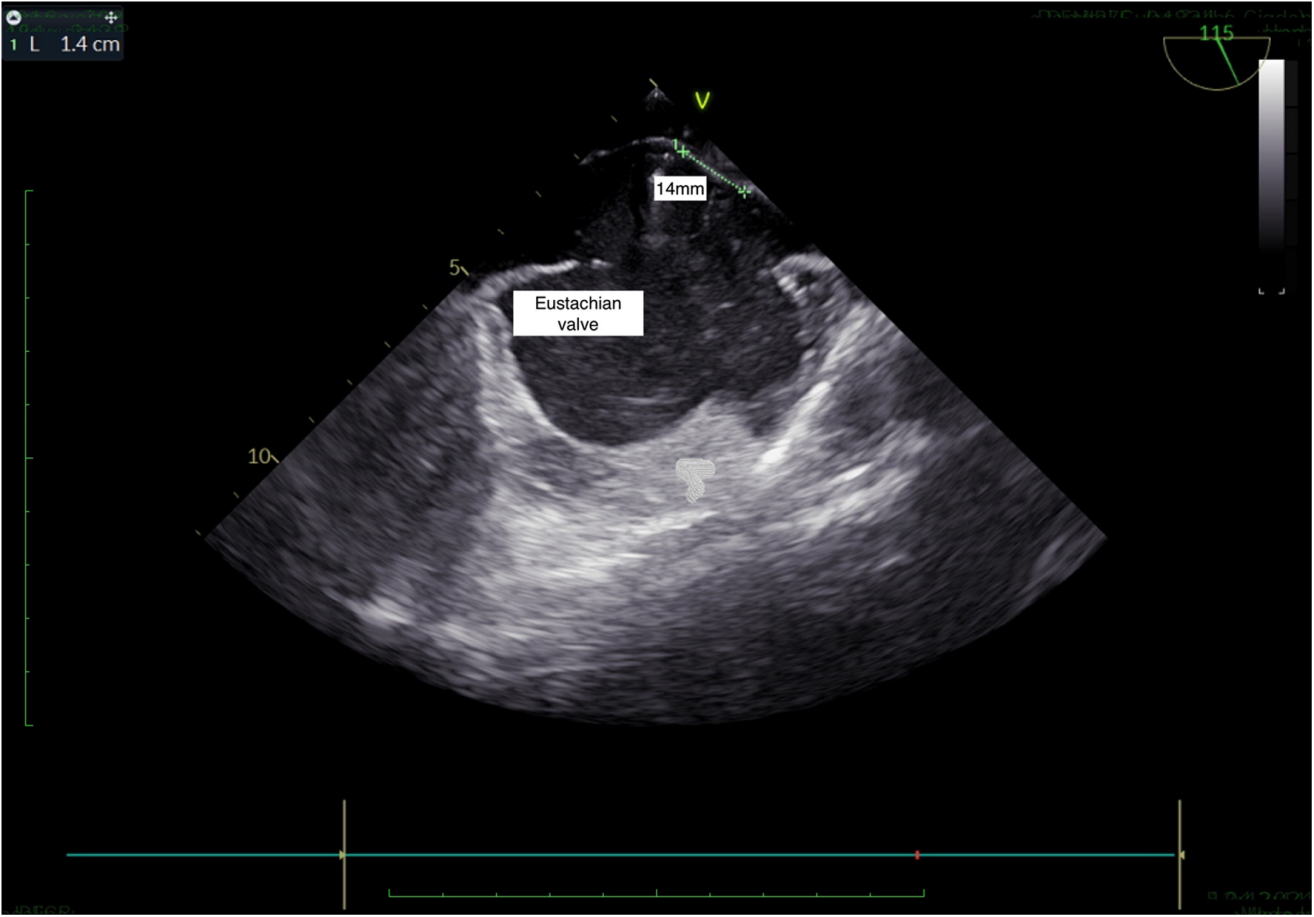
TEE, bicaval view: presence of Eustachian valve and PFO.

**Figure 5:**
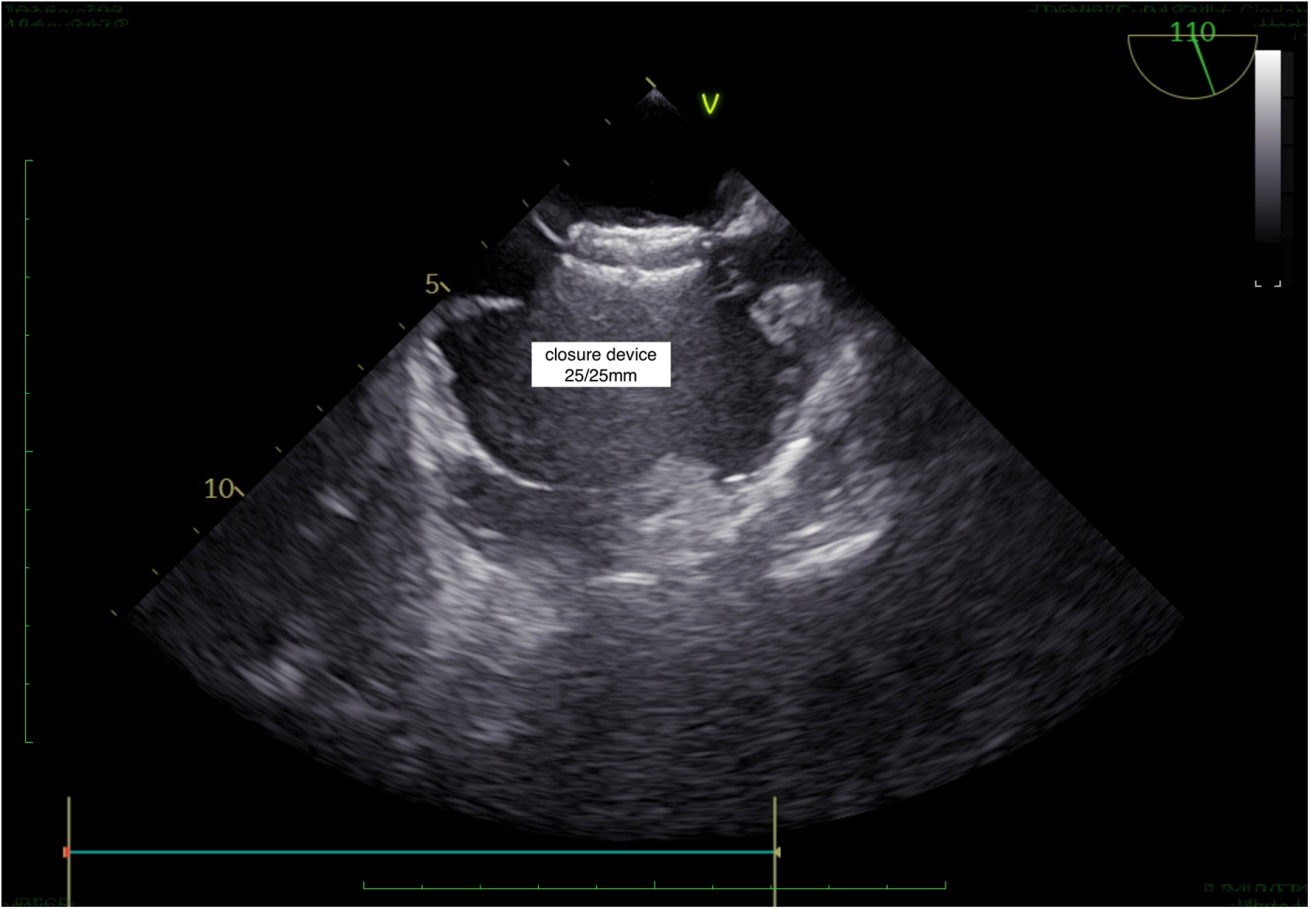
TEE, successful PFO closure.

If the septostomy point is determined by taking both A-P and bicaval sections, it provides a more accurate anatomical assessment, allowing for the correctly sized closure device to be selected. When the septostomy point was made inferiorly, the correct device size was selected without increasing the overall device size, ensuring proper closure and reducing the risk of a residual shunt (Figures 1A, 2–5). As shown in Figure 1B, in the A-P section, the distance between the septostomy point and the tip of the PFO is 8 mm, whereas in the bicaval section of the same patient, this distance is 13 mm (Figures 1C, 2–5). If the device had been selected by taking images from a single section (A-P), a small device would have been selected and a residual shunt would have been left.

If a septostomy is performed using images from only a single view (anterior-posterior or bicaval), the distance between the septostomy point and the tip of the PFO may not be accurately measured. This can lead to the selection of an incorrectly sized device, which might not fully close the PFO, resulting in a residual shunt. To avoid this issue, it is essential to use multiple views during imaging to ensure accurate measurement and appropriate device selection, which reduces the risk of residual shunts.

### Follow-up

All the patients were followed up for 12–108 months (mean follow-up 50 ± 8 months). Clinical evaluations were performed at 24 h, 1 month, 6 months, and 1 year after the procedure. Contrast echocardiography was performed on all patients to check for a right-to-left shunt. Atrial fibrillation was evaluated using a 24-h Holter monitor at 1 year post-procedure and was then subsequently conducted based on symptoms such as palpitations. In addition, all patients were monitored for the occurrence of new strokes or TIA.

All patients were followed up with clinical evaluations during the first year after the intervention. In the subsequent years, 140 patients continued with clinical evaluations, while 4 patients were followed up via telephone to assess their condition.

### Statistical analyses

SPSS (Statistical Package for the Social Sciences, version 17.0, SPSS Inc., Chicago, IL, USA) was used for statistical analyses.

The results of the patients were expressed using descriptive statistics (frequencies, means and standard deviations, and range). A *p*-value <0.05 was used to determine statistical significance.

## Results

The demographic data, clinical histories, risk factors, and laboratory findings of the patients were collected (83 women, 61 men; mean age 40 ± 10.5 years) (Table 1).

**Table 1:** The demographic data, clinical history, atrial septal anatomy, and indication for PFO closure of the participants (*n* = 144).

Mean age (±SD) (years)	40 ± 10.5
Male sex %	42% (61)
Diabetes mellitus	3% (4)
Hypertension	12% (18)
Smoking	30% (44)
Long-tunnel type	100% (144)
Body mass index (±SD) (kg/m^2^)	25.4 (±4)
Floppy interatrial septum	68% (98)
Stroke before procedure	62% (90)
A transient ischemic attack before procedure	38% (54)
Atrial fibrillation during follow-up	2%
Recurrent embolic events (due RS) during follow-up	1.4% (2)

RS, residual shunt.

All of the patients had a >10 mm long-tunnel PFO. A floppy interatrial septum (highly mobile) was detected in 62 cases (43%). A prominent Eustachian valve was present in 47 cases (33%).

The procedural success was very high (100%). However, after the procedure, moderate pericardial effusion developed in one patient (0.7%) and it was treated medically. In addition, in the first month, an acute pulmonary embolism caused by femoral vein thrombosis was observed in one patient, which was resolved with successful medical therapy (0.7%).

After the procedure, a 24-h agitated saline contrast study was performed for all patients. Nine out of 144 patients (6.2%) exhibited a right-to-left atrial shunt, and among these, 6 patients had interatrial septal hypertrophy.

In the nine patients with a shunt, we observed flow corresponding with septal movement through the occluder device discs that was associated with the cardiac cycle. By the sixth month, seven out of the nine patients showed no residual shunt, likely due to the endothelialization of the closure device. In the remaining two patients, shunts were only detected with the Valsalva maneuver, indicating an uncovered PFO. Despite this, none of these patients experienced a stroke or TIA. Consequently, the residual shunt rate after PFO closure was 1.4% at the 6-month follow-up.

In the first month, all patients were questioned about palpitations, and three patients reported brief episodes of palpitations. We conducted 24-h Holter monitoring on all patients in the first year after the intervention, and no atrial fibrillation episodes were detected. However, we assumed a 2% rate of undocumented arrhythmias. In the following years, if patients experienced palpitations, 24-h Holter monitoring was repeated.

## Discussion

Currently, there are two main techniques described for PFO closure, namely, the tunnel technique (standard) and the transseptal technique (3). The standard technique is the most common technique for PFO closure. However, it has many disadvantages in patients with a long-tunnel PFO due to the possibility of asymmetrical device placement. The device displacement may cause a residual right-to-left shunt, thrombus formation, and incomplete endothelialization at follow-up. In the transseptal technique, the septum primum is punctured to create a new hole and the device is passed through this artificial defect (4). The controlled iatrogenic defect can form a small fenestration close to the opening of the PFO tunnel. When the closure device is deployed within this fenestration to cover the PFO tunnel, a complete closure is expected. The transseptal technique has been specifically proposed for patients with a long-tunnel PFO (4–6). Moreover, the transseptal technique has also been used in complex PFOs including those with a substantial Eustachian ridge or an overly redundant Chiari network (11).

Tande et al. compared the standard technique with the transseptal puncture technique in patients with a PFO. They reported that the transseptal technique increased persistent interatrial shunting in patients with a PFO (12). However, they used the transseptal technique in only 12 patients while the standard technique was used in 108 patients. Further, the authors stated that whether the difference was caused by the techniques, devices, or the patients’ anatomies was unclear. This study had a small number of transseptal patients. In another comparative study including 22 patients with the transseptal technique and 60 patients with the standard technique in the Republic of Korea, the transseptal puncture technique was shown to result in a higher incidence of residual shunt and ischemic events. The authors stated that the transseptal technique should only be considered in specific patients as the last option (3). However, this comparative study had a small number of patients and single-plane measurement was used in TEE, similar to the standard tunnel technique. In the most recent publication, Shin et al. reported that the clinical outcomes for patients with an uncrossable PFO were similar to the patients who underwent successful PFO closure with the tunnel technique (6). In that study, patients who underwent a septostomy were excluded from the study. No difference was found in terms of stroke and TIA between the groups (the tunnel technique group and the uncrossable medical follow-up group). This finding led us to reconsider the benefit of PFO closure by passing through the tunnel.

Through clinical observation, we understood the disadvantages of the standard technique and the classic transseptal technique for patients with a long-tunnel PFO, a prominent Eustachian valve, and a mobile interatrial septum.

Furthermore, patients at high risk, including those with a prominent Eustachian valve or a mobile interatrial septum, were excluded in the aforementioned studies. Moreover, a residual shunt is found in up to 25% of patients after a PFO closure with the standard technique.

Therefore, in addition to the classic transseptal technique, the standard (tunnel) technique has also been questioned.

We realized that the classic transseptal technique measurements defined in the literature also had some restrictions. The measured distance between the puncture site and the entry/exit of the PFO channel was on a single plane, similar to the tunnel technique. Therefore, we thought that a PFO defect would not be completely covered in the event of inappropriate device size selection. Furthermore, the patient population who have undergone PFO closure with the transseptal technique was low.

The detailed transseptal technique has been routinely used for the closure of PFOs in our cardiology clinic. We use the detailed transseptal technique under the guidance of bicaval and anterior-posterior imaging for tunnel entry and exit in two projections of TEE imaging. In this study, we showed that the detailed transseptal technique for the closure of PFOs was safe and effective when performed by experienced operators. Our study had 144 patients. To our knowledge, this is the largest study to evaluate the closure of PFOs using the transseptal technique. The population encompassed patients with cryptogenic stroke. The success rate of our technique was 100%. It had low postoperative complications, which included perforation and cardiac tamponade (<1%).

The transcatheter closure technique still has some important post-procedure complications, including new-onset AF and an RS. The incidence of new-onset AF post-PFO closure varies from 3% to 14% (7). One of the possible causes for this is mechanical irritation by the device, which leads to left atrium or right atrium reentry circuits. A second cause might be a local inflammatory response as a result of a foreign body reaction. In long-tunnel PFOs especially, the tunnel technique tends to distort the septal anatomy. This condition could increase the possibility of device-related AF (1). In our study, we did not observe any atrial fibrillation episodes following the PFO closure. We accepted an undocumented arrhythmia rate of 2%.

The reason for an RS is the presence of an uncovered PFO defect due to incomplete endothelialization (13, 14). The presence of an RS after PFO closure was associated with an increased incidence of recurrent stroke or TIA (15). Incomplete sealing of a PFO should be taken into consideration in the management of recurrent stroke patients even after a technically successful PFO closure. In clinical practice, a residual shunt has been found in up to 25% of patients after a PFO closure (9). Moon et al. evaluated residual shunts with serial follow-up bubble contrast transesophageal echocardiography after PFO closures, and they found significant residual shunts in 11% of the patients at a 9-month follow-up (8). Wintzer-Wehekind et al. found that an RS was observed in 3.3% of the patients at follow-up echocardiography (16).

Contrary to these reports, we found a significant RS in two patients (1.4%) during follow-up after the closure of a PFO. We punctured the atrial septum at the junction point of the septum primum and septum secundum. The distinguishing characteristic of our technique is the determination of the optimal puncture point and the distance between the puncture point and the opening of the PFO into the left atrium. We measured the distance from the puncture point to the opening of the PFO on the left atrial side. We evaluated the defect on two planes (bicaval and anterior-posterior projection) with transesophageal echocardiography. An optimal device size was selected according to the distance between the septal puncture point and the entry of the PFO on the left atrial side. The device was deployed according to this measurement. Due to this technique, an RS developed in only two patients in the study population (residual shunts: 1.4%). This means that obtaining full anatomical closure after the appropriate device prevents the development of a residual shunt.

The correct evaluation of the patient with PFO is of paramount importance. Furthermore, it is important to select an appropriately sized device that covers both sides of the PFO tunnel. An optimal device size should be selected according to the distance between the septal puncture point and the entry of the PFO on the left atrial side. Finally, this technique should be implemented by experienced operators in experienced centers to decrease the risk of potential complications.

Transseptal puncture tools increase the cost of the procedure and require significant operator experience. In our clinic, we found that the procedural time was similar between the standard technique and the more detailed transseptal technique. However, the primary goal of PFO closure, particularly in high-risk patients (e.g., those with a long-tunnel PFO), is to reduce the residual shunt rate. Therefore, an effective method for these patients is essential. We believe that the cost-benefit ratio of this technique is acceptable. In addition, intracardiac echocardiography (ICE) can be used effectively in PFO closure, similar to TEE (17–19). However, ICE is unfortunately not available in our country.

### Limitations

This study was not a comparative study with the tunnel technique. Because of the lack of a control group and multivariate analysis to control for confounding variables and variability in follow-up, the claims of the superiority of the detailed transseptal technique were not fully substantiated.

## Conclusion

We showed that the detailed transseptal technique was safe and effective for PFO closure. PFO closure with the detailed transseptal technique significantly reduced the risk of atrial fibrillation. We found that the incidence of a residual shunt was very low following the closure of a PFO with the detailed transseptal technique. Patient selection, correct procedural measurement, and suitable device selection are key to the success of this technique. Comparative studies are needed to evaluate the efficacy and outcomes of the standard puncture technique vs. the more detailed transseptal technique for PFO closure.

## Data Availability

The datasets presented in this article are not readily available because of ethical board restrictions. Requests to access the datasets should be directed to saricamersin@yahoo.com.

## References

[1] Vitarelli A. Patent foramen ovale: pivotal role of transesophageal echocardiography in the indications for closure, assessment of varying anatomies and post-procedure follow-up. Ultrasound Med Biol. (2019) 45(8):1882–95. 10.1016/j.ultrasmedbio.2019.04.015 31104864

[2] Sperlongano S Giordano M Ciccarelli G Bassi G Malvezzi Caracciolo D'Aquino M Del Giudice C Advances in percutaneous patent foramen ovale closure: from the procedure to the echocardiographic guidance. J Clin Med. (2022) 11(14):4001. 10.3390/jcm11144001 35887765 PMC9319304

[3] Moon J Kang WC Kim S Kim MG Oh PC Park YM Comparison of out- comes after device closure with transseptal puncture and standard technique in patients with patent foramen ovale and ischemic events. J Interv Cardiol. (2016) 29:400–5. 10.1111/joic.12296 27282763

[4] Ruiz CE Alboliras ET Pophal SG. The puncture technique: a new method for transcatheter closure of patent foramen ovale. Catheter Cardiovasc Interv. (2001) 53:369–72. 10.1002/ccd.1183 11458416

[5] Thompson AJ Hagler DJ Taggart NW. Transseptal puncture to facilitate device closure of “long-tunnel” patent foramen ovale. Catheter Cardiovasc Interv. (2015) 85(6):1053–7. 10.1002/ccd.25723 25380406

[6] Shin Y Jang AY Won Y Yang T Kim J Lee J Long-term clinical outcomes for patients with uncrossable patent foramen ovale. Front Cardiovasc Med. (2023) 10:1249259. 10.3389/fcvm.2023.1249259 37900574 PMC10611517

[7] Collado FMS Poulin MF Murphy JJ Jneid H Kavinsky CJ. Patent foramen ovale closure for stroke prevention and other disorders. J Am Heart Assoc. (2018) 7(12):e007146. 10.1161/JAHA.117.007146 29910192 PMC6220531

[8] Moon J Kim M Oh PC Shin DH Park HM Jo YY Residual shunt after patent foramen ovale device closure in patients with cryptogenic stroke: serial bubble contrast transesophageal echocardiography data. J Stroke Cerebrovasc Dis. (2019) 28(2):347–53. 10.1016/j.jstrokecerebrovasdis.2018.10.006 30396838

[9] Liu TT Jiao RH Chen T Jiang ZA Bai WL. A systematic review and meta-analysis of the association between residual shunts after patent foramen ovale closure and long-term cerebrovascular events. Cerebrovasc Dis. (2023) 52(4):387–92. 10.1159/000527457 36882039

[10] Kleindorfer DO Towfighi A Chaturvedi S Cockroft KM Gutierrez J Lombardi-Hill D 2021 Guideline for the prevention of stroke in patients with stroke and transient ischemic attack: a guideline from the American Heart Association/American Stroke Association. Stroke. (2021) 52(7):e364–467. 10.1161/STR.0000000000000375 34024117

[11] Vitarelli A Mangieri E Capotosto L Tanzilli G D’Angeli I Toni D Echocardiographic findings in simple and complex patent foramen ovale before and after transcatheter closure. Eur Heart J Cardiovasc Imaging. (2014) 15(12):1377–85. 10.1093/ehjci/jeu143 25139906

[12] Tande AJ Knickelbine T Chavez I Mooney MR Poulose A Harris KM. Transseptal technique of percutaneous PFO closure results in persistent interatrial shunting. Catheter Cardiovasc Interv. (2005) 65(2):295–300. 10.1002/ccd.20377 15880797

[13] Susuri N Obeid S Ulmi M Siontis GCM Wahl A Windecker S Second transcatheter closure for residual shunt following percutaneous closure of patent foramen ovale. EuroIntervention. (2017) 13(7):858–66. 10.4244/EIJ-D-17-00061 28437244

[14] Shah AH Osten M Benson L Alnasser S Bach Y Vishwanath R Incidence and outcomes of positive bubble contrast study results after transcatheter closure of a patent foramen ovale. JACC Cardiovasc Interv. (2018) 11(11):1095–104. 10.1016/j.jcin.2018.03.008 29880106

[15] Deng W Yin S McMullin D Inglessis-Azuaje I Elmariah S Hung J Residual shunt after patent foramen ovale closure and long-term stroke recurrence: a prospective cohort study. Ann Intern Med. (2020) 172(11):717–25. 10.7326/M19-3583 32422058 PMC10021023

[16] Wintzer-Wehekind J Alperi A Houde C Côté JM Asmarats L Côté M Long-term follow-up after closure of patent foramen ovale in patients with cryptogenic embolism. J Am Coll Cardiol. (2019) 73(3):278–87. 10.1016/j.jacc.2018.10.061 30678757

[17] Rigatelli G Pedon L Zecchel R Dell'Avvocata F Carrozza A Zennaro M Long-term outcomes and complications of intracardiac echocardiography-assisted patent foramen ovale closure in 1,000 consecutive patients. J Interv Cardiol. (2016) 29(5):530–8. 10.1111/joic.12325 27500752

[18] Alqahtani F Bhirud A Aljohani S Mills J Kawsara A Runkana A Intracardiac versus transesophageal echocardiography to guide transcatheter closure of interatrial communications: nationwide trend and comparative analysis. J Interv Cardiol. (2017) 30(3):234–41. 10.1111/joic.12382 28439973 PMC5568113

[19] Lan Q Wu F Ye X Wang S Zhong J. Intracardiac vs. Transesophageal echocardiography for guiding transcatheter closure of interatrial communications: a systematic review and meta-analysis. Front Cardiovasc Med. (2023) 10:1082663. 10.3389/fcvm.2023.1082663 37215547 PMC10198467

